# Virus-specific T cells in pediatric renal transplantation

**DOI:** 10.1007/s00467-020-04522-6

**Published:** 2020-03-27

**Authors:** Thurid Ahlenstiel-Grunow, Lars Pape

**Affiliations:** Department of Pediatric Kidney, Liver and Metabolic Diseases, Hannover Medical School, Carl-Neuberg-Strasse 1, 30625 Hannover, Germany

**Keywords:** BK polyomavirus, Cytomegalovirus, Epstein-Barr virus, Adenovirus, Virus-specific T cells, Kidney transplantation, Pediatric transplantation, Immunosuppression, Viral infections, Prognostic marker

## Abstract

After pediatric kidney transplantation, immunosuppressive therapy causes an increased risk of severe viral complications, especially from cytomegalovirus (CMV), BK polyomavirus (BKPyV) or Epstein-Barr virus (EBV), and less frequent from adenovirus (ADV). However, suitable predictive markers for the individual outcome of viral infections are missing and the therapeutic management remains a challenge to the success of pediatric kidney transplantation. Virus-specific T cells are known for controlling viral replication and there is growing evidence that virus-specific T cells may serve as a prognostic marker to identify patients at risk for viral complications. This review provides an overview of the usability of virus-specific T cells for improving diagnostic and therapeutic management of viral infections with reference to the necessity of antiviral prophylaxis, timing of pre-emptive therapy, and dosing of immunosuppressive medication after pediatric kidney transplantation. Several studies demonstrated that high levels of virus-specific T cells are associated with decrease of virus load and favorable outcome, whereas lack of virus-specific T cells coincided with virus-induced complications. Accordingly, the additional monitoring of virus-specific T cells aims to personalize the management of antiviral therapy, identify overimmunosuppression, and avoid unnecessary therapeutic interventions. Prospective randomized trials in pediatric kidney recipients comparing standard antiviral and immunosuppressive regimens with T cell-guided therapeutic interventions are needed, before monitoring of virus-specific T cells is implemented in the routine care of pediatric kidney graft recipients.

## Introduction

After pediatric kidney transplantation, the immunosuppressive treatment disturbs the individual balance between virus replication and cellular immune response resulting in an elevated incidence of severe viral complications. Post-transplant primary infections or reactivations, especially by cytomegalovirus (CMV), BK polyomavirus (BKPyV) or Epstein-Barr virus (EBV), and less frequent by adenovirus (ADV), are associated with increased morbidity, mortality, and graft failure, for example, CMV disease [1], BKPyV-associated nephropathy (BKPyVAN) [2], and EBV-associated post-transplant lymphoproliferative disease (PTLD) [3]. The outcome of post-transplant viral infections is individually different, but prognostic markers are missing. Virus DNA and serology are the actual diagnostic standard to use for steering immunosuppressive and antiviral therapy in the case of primary infections or reactivations but they are insufficient to precisely predict the individual risk of viral complications. An antiviral prophylaxis or pre-emptive therapy is often recommended especially for CMV, but antiviral medication should be restricted to patients with an elevated risk of viral disease because of the high costs and severe side effects [4]. If antiviral drugs are not available, a pre-emptive reduction of immunosuppressive therapy is often performed in case of post-transplant viremia to avoid viral complications, especially for BKPyV [5] or for EBV [6], but on the other hand, it is associated with an increased risk of underimmunosuppression and rejections. Because of lack of prognostic markers, it is actually difficult to limit therapeutic interventions to patients with risk of viral complications like BKPyVAN and PTLD. Therefore, the necessity of antiviral prophylaxis, the timing of pre-emptive antiviral therapy, and the optimal dosing of immunosuppressive therapy remain a subject for debate opening a window for new biomarkers that could help to differentiate between patients with or without a need of therapeutic intervention as antiviral medication and/or reduction of immunosuppressive therapy to prevent viral complications.

Virus-specific T cells have been shown to play a significant role in control of virus replication [7]: Virus-specific CD4-positive T cells detect viral epitopes which are presented on major histocompatibility complex (MHC) class II molecules on antigen-presenting cells such as B lymphocytes, dendritic cells and macrophages, and CD8-positive T cells locate and destroy virus-infected cells which present viral antigens by MHC class I molecules. Several studies, mainly in adults, have shown that the number of virus-specific T cells is associated with the risk of virus-specific complications [8–19]. Therefore, prophylaxis, diagnosis, and treatment of viral infections after kidney transplantation may be improved by the implementation of virus-specific T cells in routine monitoring [7]. There is increasing evidence that virus-specific T cells mirror not only the virus-specific but also the general cellular immune defense. Thus, they might be additionally used for steering of the intensity of immunosuppressive treatment to avoid overimmunosuppression [7, 20]. This review will summarize the current knowledge regarding the utility of virus-specific T cells as a diagnostic tool after pediatric kidney transplantation. Currently, there is additional knowledge on diagnostic procedures and treatment with virus-specific T cells in human stem cell transplantation. This review is limited to kidney transplantation although insights from stem cell transplantation that can be transferred to solid organ transplantation are also included in the therapy section.

## Methods for detection and quantification of virus-specific T cells

A number of different assays are currently available for the detection of the cellular response to viral antigens [7], the main ones being the enzyme-linked immunospot (ELISpot) assay, the enzyme-linked immunosorbent assay (ELISA), intracellular cytokine staining followed by fluorescence-activated cell sorting (FACS) analysis, and MHC multimer staining. ELISpot, ELISA, and FACS assays are based on stimulation of virus-specific T cells followed by induction of activation markers. For stimulation, antigens such as virus-infected cell lysates, virus particles, proteins, or peptides are used. The easiest methodology is the ELISA, where cytokines such as interferon y can be measured in the supernatant of stimulated cells, and the ELISpot assay where interferon y is locally captured in mircrotiter plates. The disadvantage of these methods is that they do not allow a subclassification of stimulated cells, i.e., in CD4- and CD8-positive cells. The use of intracellular cytokine staining followed by flow cytometry overcomes this drawback and allows a complete sub-characterization of virus-specific T cells, but comes at the price of a longer and more difficult methodology. In contrast, MHC multimer staining is rapid and independent of stimulation but has the disadvantage that special MHC/peptide complexes have to be manufactured for each antigen and MHC allele so that this test is expensive and cumbersome, making it unfeasible for use in routine care [21].

There are two easy to apply assays which are commercially available for measuring the cellular response to CMV viremia but they do not directly determine the number of virus-specific T cells: QuantiFERON CMV, which is a whole blood interferon-gamma release assay based on ELISA technology [22] and T-Track CMV, which is an ELISpot assay [23]. Both assays have been used in initial diagnostic trials to determine diagnostic cutoff values [24, 25]. These assays are simple to use but both have the limitation that they only give a rough estimation of T cell activation. An analysis on the cellular level is not possible. For QuantiFERON CMV, it has been shown that it might provide false-negative results if compared with flow cytometry analysis [10]. And in a comparison between both tests, “T-Track CMV” performed better than “QuantiFERON CMV” [25]. To date, neither test has been investigated in children so it is therefore difficult to rate their diagnostic value for pediatric kidney recipients. Comparable assays are not manufactured for other viruses.

## Cytomegalovirus-specific T cells

CMV infections and CMV reactivations belong to the most common viral complications after kidney transplantation and can lead to severe morbidity by generalized CMV disease and to impairment of graft function [1]. The risk assessment for CMV-associated complications is made according to the pre-transplant CMV serostatus of recipient and donor. For pediatric kidney transplantation, the international consensus guidelines recommend the use of antiviral prophylaxis with (val-)ganciclovir for 3–6 months in the case of a seropositive donor and/or seropositive recipient; in seropositive recipients, pre-emptive therapy is considered as an alternative [26], but this medication has severe side effects such as neutropenia and nephrotoxicity [27]. However, CMV serology and DNA load are insufficient to predict the individual course of CMV DNAemia and the risk of CMV-associated complications. CMV-specific T cells control virus replication and preliminary studies have already found that the risk of post-transplant CMV-induced disease correlated with the individual number of CMV-specific T cells. Reduced frequencies of CMV-specific T cells in transplant recipients are associated with increased incidence of infectious complications [14, 28–30]. It was proven that after adult kidney transplantation, symptomatic CMV reactivations are preceded by a decrease in CMV-specific CD4 T cells frequencies and an increase in CMV load [30]. Gamadia et al. determined the kinetics and characteristics of CMV-specific T cells in the course of primary CMV infections in adult renal transplant recipients. In asymptomatic individuals, the CMV-specific CD4 T cells response preceded CMV-specific CD8 T cells response, whereas in symptomatic individuals, the CMV-specific effector memory CD4 T cell response was delayed and only detectable after antiviral therapy [31, 32]. The number of CMV-specific T cells before and after transplantation correlated with the risk of post-transplant CMV-associated events and DNAemia [33, 34]. This was also true for patients receiving anti-thymocyte globulin induction therapy [35]. Interestingly, in patients treated with the mammalian target of rapamycin (mTOR) inhibitor everolimus, the CMV-specific T cell response was more robust as compared with standard immunosuppression [36]. In immunocompetent individuals, CMV-specific T cells are induced at onset of primary infection and persist lifelong, whereas those without CMV infection do not show any specific cellular immunity. Usually, CMV-specific T cells correlate well with CMV serology [37], but in the case of unclear CMV-serostatus, analysis of CMV-specific T cells provides a reliable alternative to determine the pre-transplant CMV infection status, especially in patients with passive humoral immunity after infusions of plasma preparations [38], or in infants with passive maternal antibodies [39]. The pre-transplant absence of CMV-specific T cells in CMV-IgG-positive patients identifies CMV-naive patients at risk of post-transplant CMV-associated complications. Recently, the reverse situation was also reported, meaning that some CMV-IgG-negative kidney recipients showed pre-transplant detection of CMV-specific T cells associated with post-transplant protection from CMV infection [8, 40, 41]. Accordingly, monitoring of CMV-specific T cells offers a superior, more reliable risk assessment of post-transplant CMV complications compared with CMV serostatus alone. In a first interventional trial using the QuantiFERON CMV assay, it was proven that CMV-specific cell-mediated immunity can be used to steer the length of antiviral therapy in the case of CMV viremia after solid organ transplantation [42].

Especially in pediatric kidney recipients, who have a significantly higher rate of CMV negativity at time of transplantation and thereby a higher risk of post-transplant primary CMV infection, pre- and post-transplant monitoring of CMV-specific T cells might become a diagnostic tool to optimize the post-transplant management of antiviral prophylaxis and therapy. However, pediatric data concerning CMV-specific T cell monitoring after solid organ transplantation are rare. Our own observational study of pediatric kidney recipients showed that symptomatic courses of CMV infections and reactivations were found in the case of low CMV-specific CD4 T cell levels, whereas children with high virus-specific CD4 T cells showed asymptomatic courses. Until now, pediatric data has only been available in abstract form. Analysis of CMV-specific CD4 T cells might help to identify patients at risk of symptomatic CMV infections/reactivations and to decide upon necessity for and duration of antiviral prophylaxis and therapy. Hence, pre- and post-transplant monitoring of CMV-specific CD4 T cells may personalize CMV management and avoid unnecessary antiviral medication in CMV-IgG-positive children with sufficient levels of CMV-specific T cells. Further studies guiding CMV prophylaxis and therapy in children using virus-specific T cells are eagerly awaited.

## Adenovirus-specific T cells

ADV infections are not uncommon after pediatric kidney transplantation but seldom lead to clinical problems [43–46], whereas morbidity is much higher after stem cell transplantation [47]. There are only a few studies available concerning ADV-specific T cells after solid organ transplantation. The levels of ADV-specific T cells were investigated in adult renal transplant recipients and healthy individuals by cytokine flow cytometry [48]. In Philadelphia, Olive et al. analyzed the ADV-specific T cell response of healthy adults by ELISpot and flow cytometry [49]. Some data on ADV-specific T cells was generated by ELISpot in a small group of children after liver transplantation [50]. In one child with ADV pneumonia, ADV-specific T cells were measured after lung transplantation and the possibility of steering antiviral therapy using ADV-specific T cells is reported [11]. In our own cohort of 37 pediatric kidney recipients aged between 1 and 17 years (median 13 years), the pre-transplant prevalence of ADV-specific CD4 T cells was 76% (data not published) without any ADV-associated complications after kidney transplantation. In accordance with CMV-specific T cell data, ADV-specific T cells were permanently detectable after primary infection and fluctuated depending on the intensity of immunosuppression. Under the strengthened immunosuppression during the initial post-transplant period, we found a temporary decrease of virus-specific T cells. After reduction of the immunosuppressive therapy, virus-specific T cells began increasing again in our cohort of pediatric kidney recipients. Regarding high prevalence in childhood, ADV-specific T cells may serve as a suitable parameter to estimate the post-transplant intensity of immunosuppression and to steer the doses of the immunosuppressive medication, as is recently examined by our multicenter, randomized controlled trial (IVIST trial) [20]. Besides the use of ADV-specific T cells in our IVIST trial, no other clinical application for ADV-specific T cell monitoring after pediatric kidney transplantation has been published to date. This might be because of the very low incidence of ADV-associated complications after solid organ transplantation in children.

## BK polyomavirus-specific T cells

After kidney transplantation, primary BKPyV infections or reactivations can lead to BKPyV-associated nephropathy (BKPyVAN) with renal malfunction and risk of graft loss [5, 51–53]. In the absence of BKPyV-specific antiviral drugs, BKPyV-DNAemia-triggered reduction of maintenance immunosuppression is currently recommended in patients with BKPyV-DNAemia [5]. However, the pathophysiology of BKPyVAN is complex and the level of BKPyV-DNA in plasma alone is insufficient to estimate the risk of onset of BKPyVAN and to decide upon the necessity for therapeutic intervention [54]. It is known that BKPyV viremia after kidney transplantation does not result inevitably in BKPyVAN. Many kidney recipients show self-limiting BKPyV viremia without therapeutic interventions [53, 55, 56]. In these cases, pre-emptive reduction of immunosuppression is not only unnecessary but also associated with an increased risk of rejection.

In contrast to BKPyV antibodies, BKPyV-specific cellular immunity seems to play an important role in controlling viral replication. A few adult studies recently observed that an increase of BKPyV-specific T cells coincided with viral clearance in kidney transplant recipients [57, 58]. Accordingly, an insufficient level of BKPyV-specific T cells seems to be a key mechanism of BKPyV-associated complications after kidney transplantation. Ginevri et al. analyzed 13 pediatric kidney recipients with BKPyV-DNAemia and confirmed that a reduction of BKPyV-DNA in plasma is associated with an increase in BKPyV-specific T cells supporting the theory that the expansion of BKPyV-specific cellular immunity has a protective role [59]. Concerning BKPyV reactivations, Costa and colleagues observed episodes of BKPyV reactivation only in patients without a BKPyV-specific cellular immune response [19] and Schachtner et al. recently demonstrated that kidney transplant recipients with loss of BKPyV-specific T cells over the pre- to post-transplant period were at increased risk of BKPyV replication [18]. In 2011 and 2014, Schachtner et al. reported in a small study group of viremic patients that kidney recipients with self-limited BKPyV reactivation developed BKPyV-specific T cells without therapeutic intervention, whereas patients with BKPyVAN showed BKPyV-specific T cells only after successful treatment [55, 56]. Moreover, our own monocentric prospective, non-interventional study including 32 viremic children after kidney transplantation showed the following result: High levels of BKPyV-specific CD4 and/or CD8 T cells predicted asymptomatic BKPyV infections with self-limiting, short-term viremia (< 120 days), whereas lack or low levels of BKPyV-specific T cells were associated with long-term viremia and florid BKPyVAN [60]. Of note, the BKPyV-specific T cell level correlated with the subsequent duration of viremia but not with the BKPyV-DNA load in plasma, highlighting the additional benefit of BKPyV-specific T cells. The detection of BKPyV-specific CD4 T cells (≥ 0.5 cells/μL) and/or CD8 T cells (≥ 0.1 cells/μL) revealed a positive predictive value of 1.0 and a negative predictive value of 0.86 for self-limiting viremia. After minimization of immunosuppressive therapy and/or switch to mTOR inhibitors, BKPyV-specific CD4 T cells increased with subsequent decrease of plasma BKPyV-DNA [60].

These data highlight the predictive value of BKPyV-specific T cells after pediatric kidney transplantation to distinguish patients with self-limiting, short-term viremia from those with long-term viremia and need of therapeutic intervention. Serving as a prognostic marker, BKPyV-specific T cells may therefore identify patients at risk of BKPyVAN and thereby individualize therapeutic interventions [61].

## Epstein-Barr virus-specific T cells

Primary EBV infections or reactivations after solid organ transplantation can lead to symptomatic EBV viremia and to the development of post-transplant lymphoproliferative disease (PTLD) [62]. It has already been shown that EBV-specific T cells can be detected in children after kidney and liver transplantation but results were not associated with clinical events in this trial [13]. In a small group of pediatric liver recipients, EBV-specific T cells were monitored during first post-transplant year by ELISpot including three patients with EBV reactivations. This prospective single center study observed an immediate decline of EBV-specific T cells after transplantation and an increase after reduction of immunosuppression [50]. In addition, EBV-specific T cells have been measured in children with PTLD after solid organ transplantation, seven of whom were kidney recipients [63]. They showed an increase during PTLD treatment and a rapid re-increase in the case of EBV viremia after PTLD and can therefore be used to estimate the individual prognosis. Unfortunately, no trials have been performed to date directing PTLD treatment based on EBV-specific T cells levels. In thoracic transplantation, it could also be demonstrated that the phenotype of EBV-specific T cells varies with the severity of infection [64]. Our own data concerning EBV-specific T cells after pediatric kidney transplantation, as yet only available in abstract form, have shown that high levels of EBV-specific CD4 T cells are associated with asymptomatic self-limiting EBV viremia, whereas lack or low levels of EBV-specific CD4 T cells are found in the case of symptomatic, long-term viremia.

## Therapy with virus-specific T cells

The transfer of engineered virus-specific T cells has increasingly been used to treat life-threatening CMV [65], EBV [66], and ADV infections [67] after stem cell transplantation. The safety and efficacy of broad-spectrum T cells as treatment for ADV, EBV, CMV, and BKPyV infections after stem cell transplantation was published by Papadopoulou et al. [68]. However, data concerning therapy with virus-specific T cells after solid organ transplantation are rare. In 2016, Roemhild and Reinke summarized the data on virus-specific T cell transfer in solid organ transplantation, mainly concentrating on therapy with EBV-specific T cells in adults [69]. After solid organ transplantation, data on therapy with EBV-specific T cells are far more frequently published compared with data for CMV-specific T cells or other viruses. In children, there are only a few case reports and small case series on EBV-specific T cell therapy for treatment of EBV-associated PTLD after kidney and liver transplantation [70, 71]. However, not even as much as a case report has been published about adoptive T cell transfer in children after solid organ transplantation with viral diseases other than EBV-associated PTLD. As this method is expensive and associated with high risks for the recipient, future reports and studies are awaited, so that the clinical utility of treatment with virus-specific T cells can be assessed for children after solid organ transplantation. It can be speculated that therapy with virus-specific T cells will be limited to children with infections that are associated with a high risk of graft loss (i.e., BKPyVAN) or life-threatening disease (i.e., PTLD) and which are resistant to any other treatment.

## Steering of immunosuppressive therapy by virus-specific T cells

Post-transplant monitoring of virus-specific T cells showed that levels of virus-specific T cells fluctuated depending on the intensity of immunosuppression. During the initial post-transplant period—at the time of very intensive immunosuppressive therapy—virus-specific T cells were decreased and showed increase after reduction of immunosuppressants [7]. Accordingly, it is hypothesized that virus-specific T cells represent not only virus specific but also general cellular immune defense and thereby correlate with the individual susceptibility to infections. Serving as a marker of overimmunosuppression, additional monitoring of virus-specific T cells might optimize steering of immunosuppressive therapy compared with blood level monitoring alone [7]. To our knowledge, our IVIST trial is the first study considering the benefit of additional steering of immunosuppressive drugs by virus-specific T cells. The study protocol of this investigator-initiated, multicenter, randomized controlled trial has been already published [20]. Sixty-four pediatric kidney recipients were randomized 4 weeks after transplantation either to a non-intervention group with classical trough level monitoring of immunosuppressants or to an intervention group with additional steering by virus-specific T cell levels against CMV, ADV, and herpes simplex virus (HSV). Regarding high prevalence in childhood (especially of ADV-specific T cells) and long-term persistency after primary infection, CMV-, ADV-, and HSV-specific T cells are suitable for post-transplant monitoring. Both groups received the same immunosuppressive regimen consisting of cyclosporine A and everolimus with the same target range of trough levels. The primary endpoint of the study is the glomerular filtration rate (GFR) 2 years after transplantation. Secondary endpoints are the number and severity of infections and the exposure to immunosuppressive drugs. In terms of an effect-related drug monitoring, the study design aims to realize a personalization of immunosuppressive management after transplantation. The results of the trial are expected in 2020 and, hopefully then, the IVIST trial will answer the question of whether the new concept of steering immunosuppressive therapy by virus-specific T cell levels leads to optimization of post-transplant management.

## Conclusion

New diagnostic strategies using markers of the individual cellular immune response such as virus-specific T cells seem to be promising in pediatric kidney transplantation to estimate the outcome of post-transplant viral infections and to decide on the necessity of antiviral medication and/or reduction of immunosuppressive therapy and thereby to avoid unnecessary therapeutic intervention. Analysis of virus-specific T cells may become an important step towards the introduction of precision medicine in pediatric kidney transplantation. The measurement of virus-specific T cells at time of onset of viremia, challenging whether a therapeutic intervention should be performed, could become a part of routine care. Prospective, interventional trials comparing standard of care with T cell-based steering of antiviral and immunosuppressive therapy in case of post-transplant viral infections are needed in order to confirm the usability of this strategy in viremic patients. Furthermore, if the strategy can be confirmed in prospective trials, virus-specific T cells might also be used as an additional routine tool to measure the intensity of immunosuppression after pediatric kidney transplantation in order to avoid overimmunosuppression.

## Abbreviations

*ADV*: Adenovirus
*BKPyV*: BK polyomavirus
*BKPyVAN*: BK polyomavirus-associated nephropathy
*CMV*: cytomegalovirus
*EBV*: Epstein-Barr virus
*ELISA*: enzyme-linked immunosorbent assay
*ELISpot*: enzyme-linked immunospot
*FACS*: fluorescence-activated cell sorting
*GFR*: glomerular filtration rate
*HSV*: herpes simplex virus
*MHC*: major histocompatibility complex
*mTOR*: mammalian target of rapamycin
*PCR*: polymerase chain reaction
*PTLD*: post-transplant lymphoproliferative disease
